# Gastrointestinal transit tolerance, cell surface hydrophobicity, and functional attributes of *Lactobacillus Acidophilus* strains isolated from Indigenous Dahi

**DOI:** 10.1002/fsn3.2468

**Published:** 2021-07-13

**Authors:** Wajiha Farid, Tariq Masud, Asma Sohail, Nazir Ahmad, S. M. Saqlan Naqvi, Sipper Khan, Amjad Ali, Salah A. Khalifa, Abid Hussain, Sartaj Ali, Maryum Saghir, Azhari Siddeeg, Muhammad Faisal Manzoor

**Affiliations:** ^1^ Department of Food Technology Pir Mehr Ali Shah, Arid Agriculture University Rawalpindi Pakistan; ^2^ Institute of Food & Home Sciences Government College University Faisalabad Pakistan; ^3^ Institute of Biochemistry and Biotechnology Pir Mehr Ali Shah, Arid Agriculture University Rawalpindi Pakistan; ^4^ Tropics and Subtropics Group Institute of Agricultural Engineering University of Hohenheim Stuttgart Germany; ^5^ Department of Agriculture and Food Technology Karakoram International University Gilgit Pakistan; ^6^ Department of Food Science Faculty of Agriculture Zagazing University Sharkia Egypt; ^7^ Department of Manufacturing Engineering National University of Science and Technology Islamabad Pakistan; ^8^ Department of Food Engineering and Technology Faculty of Engineering and Technology University of Gezira Wad Medani Sudan; ^9^ School of Food and Biological Engineering Jiangsu University Zhenjiang China; ^10^ Riphah College of Rehabilitation and Allied Health Sciences Riphah International University Faisalabad Pakistan

**Keywords:** antioxidants, *bsh* gene amplification, cell surface hydrophobicity, cellular auto‐aggregation, exopolysaccharide (EPS), gastrointestinal transit tolerance, hypocholesterolemic activity

## Abstract

Strains of *Lactobacillus acidophilus* WFA1 (KU877440), WFA2 (KU877441), and WFA3 (KU877442) were isolated from indigenous Dahi (yogurt), screened, and selected based on acid and bile tolerance along with the antimicrobial activity. These selected strains were further assessed for their probiotic and functional attributes. Results for simulated gastric and intestinal tolerance/ resistance revealed that all three strains can resist and survive under the following mentioned conditions. To access cell surface hydrophobicity, bacterial adhesion to hydrocarbons (BATH), cellular auto‐aggregation, and salt aggregation were performed. In BATH, adhesion of strains against three hydrocarbons namely xylene, dichloromethane, and hexadecane was conducted. The results show that strains showed the least adhesion to xylene (54.25%) as compared to dichloromethane (55.25%) and hexadecane (56.65%). WFA1 showed maximum adherence percentage (55.48%) followed WFA2 (55.48%) and WFA3 (51.38%). Cellular auto‐aggregation varied from 21.72% to 30.73% for WFA3 and WFA1, respectively. In the salt aggregation test (SAT), WFA1, WFA2, and WFA3 aggregated at 0.6, 1.0, and 2.0 molar concentrations of ammonium sulfate, respectively. PCR amplification of bile salt hydrolase gene (bsh) was performed and sequences were submitted to the public database of NCBI and Gene bank under accession numbers, KY689139, KY689140, and KY689141. Additionally, a cholesterol‐lowering assay was conducted and up to 26% reduction in cholesterol was observed by the strains. Regarding functional properties, exopolysaccharide (EPS) production, and antioxidant potential, strain WFA1 showed promising results EPS (1.027mg/ml), DPPH (80.66%), ABTS (81.97%), and reducing power (1.787). It can be concluded from the present study that the mentioned strains of *L. acidophilus* (WFA1, WFA2, and WFA3) are strongly hydrophobic; thus having an ability to survive and colonize under the gastrointestinal tract which confirms their probiotic nature. Regarding their functional properties, *L. acidophilus* WFA1 (KU877440) showed excellent properties of antioxidants and EPS production.

## Graphical abstract

Strains of *L. acidophilus* (WFA1, WFA2 and WFA3) have resisted and survived under GI tract conditions, furthermore; they have the capability to adhere and colonize in the intestinal cavity, thereby confirming their probiotic potential. These strains also have cholesterol‐lowering property along with high EPS production and antioxidant activity. Among the three strains, WFA1 was selected further due to its potential functional properties and was supplemented to produce probiotic fermented milk.

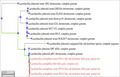

## 1 INTRODUCTION

The presence of *L. acidophilus* is reported in indigenously produced fermented milk products locally named as “dahi” of Pakistan, along with other members of lactic acid bacteria (LAB), *L. acidophilus* is promising, completely characterized, well‐documented probiotic member of LAB (Behbahani et al., 2019) and is already included in the generally recommended as safe (GRAS) category. According to FAO (Food and Agriculture Organization, 2006), “Probiotics are the live microorganisms which when administered in an adequate amount, confers health benefits to the host.” They are also categorized into a category of nutraceutical (Hill et al., 2014).

The most important criterion for probiotics is to remain viable under harsh environmental conditions of the gastrointestinal tract. Low pH in the stomach and gastric enzymes along with the presence of bile and intestinal enzymes in the intestine is considered fatal for bacterial strains. So for the selection of probiotics, strains have to access acid, bile, and digestive enzyme tolerance and gastrointestinal (GI) transit tolerance. Furthermore, they must possess the quality of intestinal colonization. This characteristic makes it the most suitable to not only remove/decrease the adherence of enteropathogens (Monteagudo‐Mera et al., 2019), but also need to exert their health benefits on their respective host.

Cell surface hydrophobicity of a probiotic strain is a measure of its intestinal colonization, that is, adhesion and persistence once they have entered the intestinal cavity. The higher the hydrophobicity, higher colonization was observed (de Souza et al., 2019). This was further resolved if there is better bacterial adhesion to hydrocarbons (BATH), cellular auto‐aggregation, and salt aggregation. BATH deals with the adhesion property of strain and both cellular auto‐aggregation and salt aggregation are clumping or self‐aggregation capacity of cells related to its persistence (Saito et al., 2019),

Probiotic has also been reported for their hypocholesterolemic activity which can be attributed to bile salt hydrolase (bsh) activity (Huang et al., 2019; Kumar et al., 2012). It is an enzymatic breakdown or deconjugation of bile salts or bile acids by probiotic LAB, increases its excretion or decrease its reabsorption (Ishimwe et al., 2015), and can be attributed to in vivo lowering of serum cholesterol levels (Patel et al., 2010).

Production of EPS by LAB is one of the most promising functional properties. EPS are naturally produced sugar polymers having a distinct and significant role, one of which is to enhance the taste and texture of food (Badel et al., 2011). Its bio‐functional property increases its importance as food‐grade hydrocolloids, emulsifiers, or bio stabilizers in fermented foods (Rühmann et al., 2015). Apart from this, EPS have also strongly antioxidant, anticancer, and anti‐inflammatory activity (Deepak, Ramachandran, et al., 2016). Along with other functional properties, yogurt bacteria were reported to have a role in reducing oxidative stress because they have a strong potential of scavenging free radicals such as reactive oxygen species (ROS), peroxide, and superoxide (Ji et al., 2015; Xing et al., 2015). Antioxidants prevent oxidative damage, thus capable of slowing down the process of aging and likewise protects the human body from the progression of different diseases.

Previously studies have confirmed the presence of *L. acidophilus* and other LAB in indigenous dahi collected from the area of Rawalpindi, Pakistan (Soomro & Masud, 2012). But their probiotic nature and functional properties were not assessed. Studies also lacked the molecular characterization. Thus, the present study was designed to assess the potential of local strains of *L. acidophilus*. Molecular characterization along with initial isolation and screening of *L. acidophilus* on the basis of acid and bile tolerance and antimicrobial activity against pathogens has already been published (Farid et al., 2016). Hence, the current paper discusses the GI transit tolerance, adhesion properties, and hypocholesterolemic activity of *L. acidophilus* strains (WFA1, WFA2, and WFA3). Furthermore, these strains were screened for exopolysaccharide (EPS) production and antioxidant activity to further uses in probiotic product development.

## 2 MATERIALS AND METHODS

In a preliminary isolation study, screening and molecular characterization of *L. acidophilus* were performed. Briefly, isolation was conducted on MRS media supplemented with 0.7% bile salts. Out of 57 strains of LAB, 18 were confirmed as *L. acidophilus* through API CHL 50. These 18 strains were further screened for acid tolerance and antimicrobial activity against different pathogens namely *Escherichia coli* ATCC 25922, *Salmonella paratyphi* ATCC 5702, *Staphylococcus epidermis* ATCC 12228, *Acinetobacter baumannii* ATCC 17978, and *Staphylococcus aureus* ATCC 29213. Strains showing higher antimicrobial activity were selected and characterized at the molecular level through polymerase chain reaction (PCR) using universal primers 9F and 1510R and sequencing, and then, their sequence was submitted to the NCBI Gene Bank as *Lactobacillus acidophilus* WFA1 (KU877440), WFA2 (KU877441) and WFA3 (KU877442) (Farid et al., 2016).

In the present study, the probiotic potential of these characterized strains was assessed including GI transit tolerance and cell surface hydrophobicity which ensures their survival and colonization in the GI tract. Furthermore, their functional properties, that is, *bsh* activity, cholesterol‐lowering ability, EPS quantification, and antioxidant properties, were also analyzed.

### 2.1 GI transit tolerance

For GI transit tolerance, the method of Zhou, Pan, Wang, and Li (2007) was followed. Firstly gastric (pH 2, 3, and 4) and small intestinal (pH 8 and 0.3% bile salts) juices were prepared with pepsin (3 g/L) and pancreatin (1 g/L) in saline solution (0.5%) respectively.

The bacterial cell pellet was harvested, washed twice, and resuspended in PBS buffer. A volume of 2 ml of gastric juice with pH 2 and 3 was taken in a sterilized Eppendorf tube containing 0.4 ml of bacterial cell suspension already mixed with 0.6 ml of saline and vortex gently before incubating at 30℃ for 5 hr. The same procedure was followed for intestinal tolerance but incubation was done for 12 and 24 hr. The plate count method of the colony‐forming unit (log CFU/ml) was followed to calculate the survival of bacterial strains.

### 2.2 Cell surface hydrophobicity

#### 2.2.1 Bacterial Adhesion to Hydrocarbons (BATH)

The BATH was adopted from the method of (Kaushik et al., 2009). Overnight the cell pellet grown culture was washed and resuspended in a phosphate urea magnesium buffer (PUM) to an absorbance of 0.7 at 600 nm. A volume of 3.0 ml of *Lactobacilli* cell was mixed with 1.0 ml of xylene or dichloromethane or n‐hexadecane and incubated at 37℃ for 10 min. After vortexing, incubation for phase separation was carried out at 37℃ for 1 hr, where the absorbance of the aqueous phase was recorded at 600 nm. The surface hydrophobicity (percent) was calculated by using the following equation:

$$\text{Surfacehydrophobacity}\left(\%\right)=\frac{\text{OD}\left(\text{initial}\right)‐\text{OD}\left(\text{final}\right)}{\text{OD}\left(\text{initial}\right)}\times 100.$$

#### 2.2.2 Cell aggregation

Cellular aggregation was conducted by Zuo et al. (2016). Bacterial cells were collected, washed, resuspended in sterilized PBS (step 1), and the concentration was adjusted to an absorbance of 0.5 at 600 nm using the respective buffer. The cell pellets were taken out and mixed with an equal volume of broth obtained at step 1. This mixture was incubated at 37℃ for 2 hr. The upper suspension was then taken to measure the absorbance (Abs final), using the broth as reference.

$$\text{Cell}\;\text{aggregation}\left(\%\right)=\frac{\text{OD}\left(\text{initial}\right)‐\text{OD}\left(\text{final}\right)}{\text{OD}\left(\text{initial}\right)}\times 100.$$

#### 2.2.3 Salt aggregation test (SAT)

Salt aggregation test assay was conducted (Nwanyanwu & Abu, 2013) in which different molarities (M) of (NH_4_)_2_SO_4_ solution (ranging from 0.2 to 4.0 M) were used and further aided by 0.1% (w/v) methylene blue solution to improve visualization. Equal volumes (100 ul) of cell suspension and (NH_4_)_2_SO_4_ were mixed and checked for the concentration on which bacterial cells were clumped or salted out (lower the concentration, higher will be the hydrophobicity). The classification was expressed as follows:

Strongly hydrophobic = < 1.0 M, Hydrophobic = 1.0–2.0 M, Hydrophilic = > 2.0 M.

### 2.3 Cholesterol lowering assay

#### 2.3.1 Bile salt hydrolase (*bsh*) activity

For *bsh* activity, overnight grown bacterial culture was streaked on MRS agar‐containing bile salts (0.5%) along with 0.37 g/L CaCl_2_ and incubated anaerobically at 37℃ for 48–72 hr. White opaque bacterial growth or presence of the precipitated hydrolyzed product of bile salts in or around the colony growth were recorded, it exhibited the indication of bsh activity (Pereira et al., 2003; Tsai et al., 2014).

#### 2.3.2 Molecular confirmation for *bsh* gene

For PCR amplification already submitted sequence of *bsh* gene of *L. acidophilus* (Accession # AF091248.3 *Lactobacillus acidophilus* putative bile salt hydrolase operon) was taken as reference and NCBI primer blast designing software was used for primer designing (Table 1).

**TABLE 1:** Sequence of forward and reverse primer for *bsh* gene amplification

Primer sequences		
Forward primer	Reverse primer	This study
5´CAGGATTATGGCGAAGGCGT3´	5´TCGAGCGGTTGACGGTATTG3´	

Deoxyribonucleic acid (DNA) template was prepared by the method described by (Plourde‐Owobi et al., 2005) with some modification. Freshly grown colonies of *L. acidophilus* were picked and transferred to 10 ul Tris‐EDTA (ethylene diamine tetra‐acetic acid) buffer (10 ml of 1.0 M Tris‐HCl buffer (pH 7.5) and 2 ml of 0.5 M EDTA solution (pH 8) make the volume up to 1 L) by using sterilized toothpicks and incubated at 80℃ for 15 min, followed by centrifugation at 12,000 rpm for 15–20 min and then the supernatant was taken as the template. The reaction mixture, its concentration, and details of the PCR cycle are given in Tables 2 and 3, respectively.

**TABLE 2:** Quantity of chemicals used in PCR amplification

Reaction mixture	Quantity (ul)
Reaction buffer	1.0
MgCl_2_	0.6
Primers	
(Reverse)	0.5
(Forward)	0.5
Taq DNA polymerase	0.4
dNTPs	0.2
DNA template	2.0
Water	4.8
Total	10.0

**TABLE 3:** Cycle conditions (temperature and time) for PCR amplification

	Temperature (^o^C)	Time (mins)	No. of cycles
Initial heating	94	2	1
Denaturation	94	1	33
Annealing	61.5	1	
Extension	72	1.5	
Final extension	72	20	1

Gel electrophoresis was performed by running agarose gel (1%) in TAE (Tris‐Acetate EDTA) buffer for about 20–30 min on 70 volts and 110 mA followed by staining in ethidium bromide solution and visualization conducted under UV transilluminator.

#### 2.3.3 Sequencing, sequence analysis, and phylogenetic tree

The amplified products were sequenced from macrogen (Korea) in sense and antisense directions. The obtained sequences were analyzed by using Bio Edit sequence alignment software and BLAST tool of the National Centre for Biotechnology Information (NCBI) (http://blast.ncbi.nlm.nih.gov/Blast.cgi). Final sequences were submitted in the public database of NCBI under accession numbers, KY689139, KY689140, and KY689141. The phylogenetic tree was constructed using 15 closely related sequences (99%–100% genetic homologies) of *bsh* gene which were taken from NCBI after BLAST.

#### 2.3.4 Hypocholesterolemic activity of *L. acidophilus* strains

MRS broth containing 0.2% bile salt (w/v) and 0.01% (w/v) cholesterol (polyoxyethanyl‐cholesteryl Sebacate; Sigma) was prepared, inoculated with overnight grown culture, and were incubated at 37℃ for 24 hr. Bacterial cells were removed after 6, 12, 18, and 24 hr of inoculation, and cholesterol contents were measured (Rudel & Morris, 1973).

### 2.4 EPS extraction and quantification

Exopolysaccharide extraction and quantification (6, 12, 18, and 24 hr of inoculation) was performed by the method described by Feldmane et al. (2013) with some modification. Overnight‐grown bacterial culture was boiled (20–30 min), cooled, and centrifuged (8,000 rpm for 10 min), followed by the addition of 17 ml of 85% trichloro‐acetic acid. Samples were cooled at 4℃ and centrifuged again. Cold ethanol (1:3) was finally used to precipitate EPS.

For quantification of EPS, an equal amount of the sample and phenol red solution (5%) were taken in a test tube (400 µl). For control, distilled water replaced the sample. Then 2 ml of concentrated sulfuric acid was added and left for 10 min, followed by shaking and incubation (30℃ for 10 min). Absorbance was recorded at 490 nm. The same procedure was followed for the preparation of the glucose concentration curve (0.2–1.0 mg/ml), taken as a standard for quantification of EPS.

### 2.5 Antioxidative assay

#### 2.5.1 Preparation of cell free supernatant (CFS)

For CFS preparation, the pH of the overnight grown culture was maintained at 5.5 (1 M NaOH) then centrifuged (8,000 rpm for 10 min). The supernatant was separated and passed through a 0.2 um syringe filter to remove bacterial cells completely.

#### 2.5.2 Antioxidant potential

Antioxidant activity or radical scavenging ability of CFS of *L. acidophilus* strains was conducted after 6, 12, 18, and 24 hr of inoculation and incubation. The maximum antioxidant activity was observed after 24 hr of incubation which were further compared with ascorbic acid (0.1%) (Water‐soluble antioxidant).

##### Determination of 2, 2‐diphenyl‐1‐picrylhydrazyl (DPPH) scavenging ability

The DPPH scavenging activity was measured according to the method of Arora and Chandra (2010). DPPH (0.1 mM) solution was prepared freshly in ethanol, 1 ml of this solution was mixed with 0.5 ml of CFS and thereafter shaken vigorously and was left to stand for 30 min in the dark. Change in color at 517 nm determines the DPPH radical scavenging activity, which was calculated according to the following equation:

$$\text{DPPH}\;\text{scavenging percent}=\left[1‐\frac{\left(\text{A1}‐\text{A2}\right)}{\text{A0}}\right]\times 100.$$

whereas,

A0 = Absorbance of control, A1 = Absorbance of CFS.

A2 = Absorbance without DPPH and broth was taken as reference.

##### Determination of 2, 2'‐azino‐bis (3 ethylbenzothiazoline 6‐sulphonic acid) ABTS cation scavenging activity

ABTS cation scavenging assay was performed according to the procedure described by Ji et al. (2015). Firstly ABTS stock solution was prepared (7 mM ABTS and 2.45 mM potassium per sulfate) and left for –416 hr and diluted to an absorbance of 0.7 at 734 nm with ethanol. 0.3 ml of CFS and 2.7 ml of ABTS solution were also mixed for 45 s and after 1 min spectrophotometric absorbance was measured at 734 nm. The following equation was used to calculate scavenging activity: $\text{Scavenging percent}=1‐\left[\text{Abs}\left(s\right)/\text{Abs}\left(c\right)\right]\times 100$.

Abs (s) = Absorbance of sample Abs (c) = Absorbance of control.

##### Reducing power

The CFS reducing the power of CFS was determined as described by Xing et al. (2015) with slight modification. A sample (CFS, MRS broth, 0.5 ml) was briefly mixed with potassium ferricyanide (1%, 0.5 ml) and PBS (pH 6.6, 0.5 ml). Subsequently, the mixture was heated at 50℃ for 20 min and allowed to cool. Upon cooling, 0.5 ml of 10% trichloroacetic acid (TCA) was added to the mixture and then centrifuged at 3,000 *g* for 5 min. The supernatant (1 ml) was mixed with ferric chloride (0.1%, 1 ml) and was allowed to react for 10 min. The absorbance of the mixture was determined at 700 nm. Higher absorbance of the mixture indicated higher reducing activity.

### 2.6 Statistical analysis

Data were analyzed statistically by software Statistix 8.1 (for ANOVA), for percentages/ comparisons and standard deviation MS‐excel was used. Likewise, for the alignment of *bsh* gene sequences, BioEdit sequence alignment software was used.

## 3 RESULTS

### 3.1 GI Transit tolerance

Gastrointestinal transit tolerance and survival of LAB under these conditions is preliminary for the selection of probiotics. Results for simulated gastric and intestinal conditions of all the three strains (WFA1, WFA2, and WFA3) are presented in Table 4. These results show their ability to resist and survive under these harsh environmental conditions. Although a decrease in log CFU/ml has been observed in both simulated gastric and intestinal conditions but to a lesser extent.

**TABLE 4:** Survival percentage of *Lactobacillus acidophilus* strains under simulated gastric (pH 2, 3, and 4) and intestinal conditions (incubation for 12 and 24 hr)

Strains	Survival % under gastric conditions	Survival % under intestinal conditions			
	pH2	pH3	pH4	12 hr	24 hr
WFA1	47.35	63.25	80.5	78.87	64.26
WFA2	50.45	62.5	78.7	69.95	57.45
WFA3	43.12	57.9	79.85	84.65	71.36

Under simulated gastric conditions, pH 2 has a more severe impact on the survival percentage of strains as compared to pH 3 where a sharp decrease was observed in log CFU/ml at this pH. At pH 2, survival of strains ranged from 43.19% to 50.45% (almost about 49.55%–56.81% reduction). At pH 4, least impact of pH was observed where the survival rate was about 78.7%–80.5% (21.3%–19.5% reduction). Similarly in simulated intestinal conditions of incubation for 24 hr showed more decrease in log CFU/ml compared to 12 hr. Survival ranges from 57.45% to 71.36% (42.55%–28.64% reduction) after 24 hr of incubation. This decrease was obvious because bacterial strains were not acclimatized to these harsh conditions but their survival ability was not much affected.

### 3.2 Cell surface hydrophobicity

#### 3.2.1 Bacterial adhesion to hydrocarbons

Bacterial adhesion to hydrocarbons measures the adhesion property or ability of LAB to adhere to the organic compounds (nonpolar). Higher the nonpolarity of surfaces, higher will be adhesion to hydrocarbons and more will be hydrophobicity.

**FIGURE 1:**
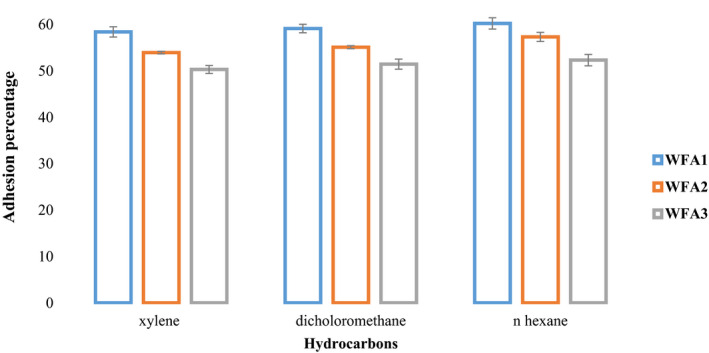
Graphical representation of the percentage of bacterial adherence to hydrocarbons of selected strains of *Lactobacillus acidophilus* by using xylene, dichloromethane, and hexadecane

The percent adhesion of strains to different hydrocarbons has been presented graphically in Figure 1. Statistical analysis revealed that both the adhesion ability of strains and adherence percentage of different hydrocarbons are different significantly. *L. acidophilus* WFA1 exhibited maximum adherence (59.28%), followed by *L. acidophilus* WFA2 (55.48%). While *L*. *acidophilus* WFA3 (51.38%) had the lowest adhering property. Among the three tested hydrocarbons, hexadecane (56.65%) showed the maximum score of adhesion followed by dichloromethane (55.25%) and xylene (54.25%).

#### 3.2.2 Cellular auto‐aggregation

It is the measure of self‐clumping or the aggregating ability of cells of bacteria which is an important criterion for its persistence and colonization in the intestine. The percent cell auto‐aggregation of different strains of *L. acidophilus* presented in Table 5 shows that there is a significant difference in the aggregation property of all three probiotic strains. The mean value of aggregation among different strains ranged from 21.72% to 30.73%. *L. acidophilus* WFA1 was found to have maximum aggregating % (30.7%) followed by *L. acidophilus* WFA2 (27.34%). *L. acidophilus* WFA3 (21.72%) has the lowest adhering property among all tested strains.

**TABLE 5:** Comparison of aggregation percentage, salt aggregation, and cholesterol‐lowering ability among selected strains of *Lactobacillus acidophilus*

Strains	Aggregation (%)	SAT	Cholesterol‐lowering (%)
WFA1	30.73 ± 1.16^a^	0.6 M	26.55 ± 0.79^a^
WFA2	27.34 ± 0.93^b^	1 M	26.22 ± 1.36^a^
WFA3	21.72 ± 0.83^c^	2 M	26.43 ± 0.63^a^

Note

The values are means ± SE; each value is expressed as a mean of three experiments, different letters in a column indicated the significant difference.

### 3.3 Salt aggregation test (SAT)

Salt aggregation test is the measurement of aggregation or precipitation of probiotic *L. acidophilus* strains in different concentrations or molecular strength of ammonium sulfate solution ranging from 0.2 to 4 M (Tyfa et al., 2015). Results for the salt aggregation test have been presented in Table 5. According to the criterion, one of *L. acidophilus* strains falls in the category of being strongly hydrophobic by agglutinating at 0.6 M (WFA1) and the other two were hydrophobic as they clumped at 1 M (WFA2) and 2 M (WFA3) of ammonium sulfate. It was observed that lower the concentration of ammonium sulfate on which aggregation was recorded, the higher will be the hydrophobicity of strain.

### 3.4 Hypocholesterolemic activity

#### 3.4.1 Bile salt hydrolase activity (*Bsh* gene amplification, sequencing, and phylogenetic tree)

**FIGURE 2:**
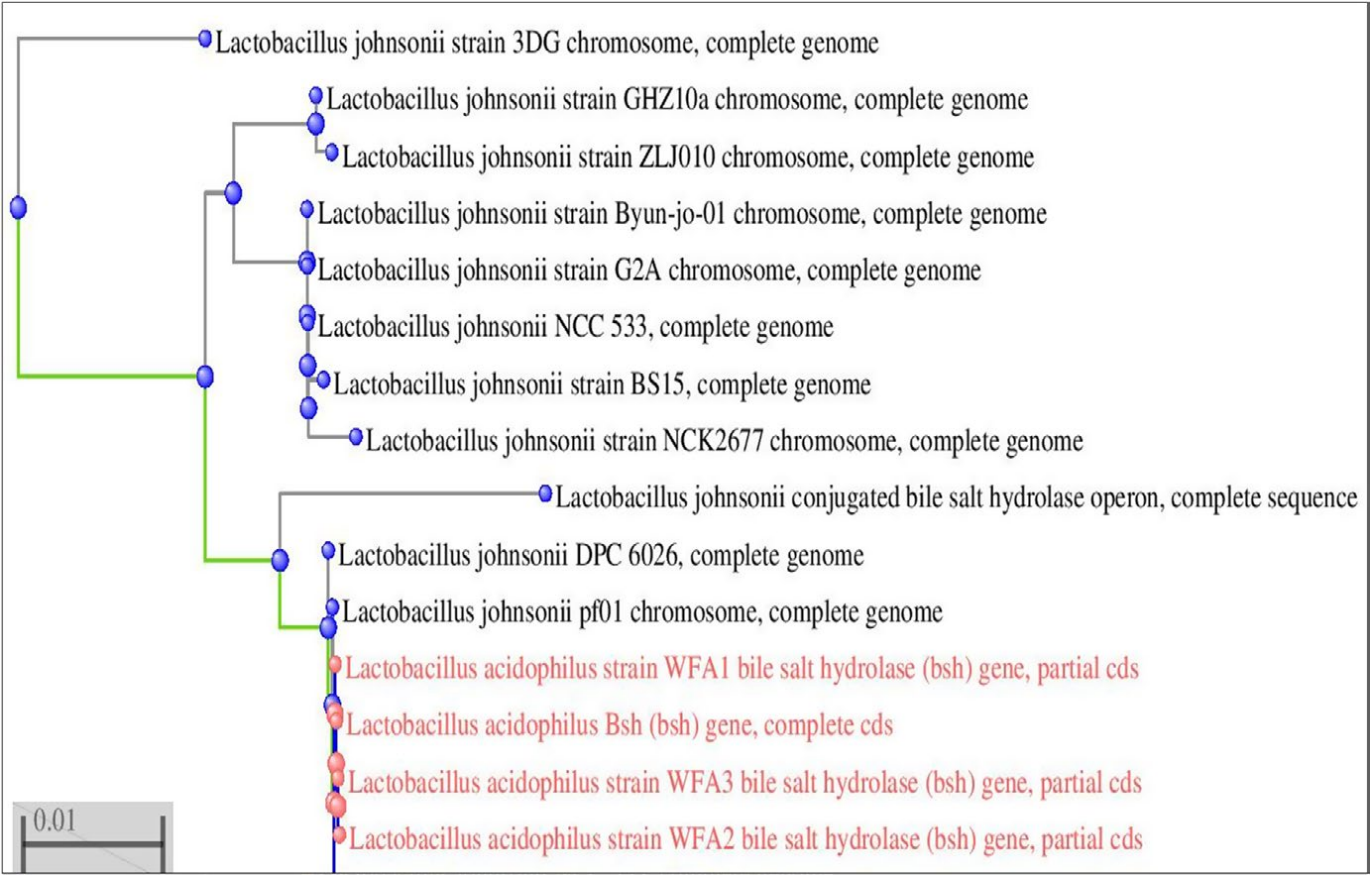
Phylogenetic tree of *Bsh* gene sequence of *Lactobacillus acidophilus* strains with the most similar/resembled sequences

**FIGURE 3:**
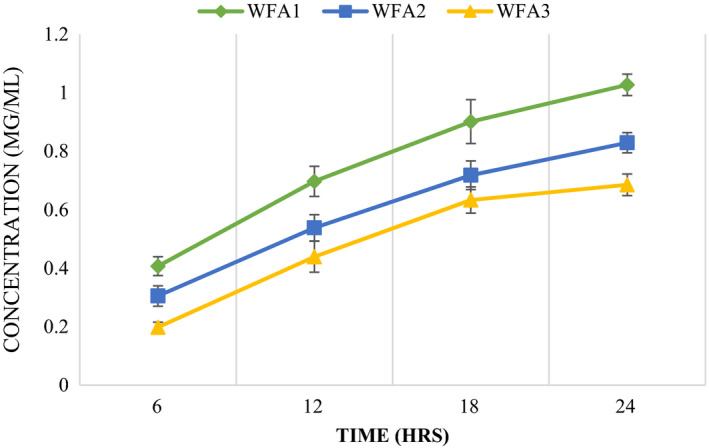
Comparative analysis of EPS quantification of *Lactobacillus acidophilus* strains at a different time interval at 37℃

All three strains showed thick white opaque colonies on differential media which confirmed their *Bsh* activity as shown in Figure 2 and were further subjected to PCR amplification of the bile salt hydrolase gene. A product of 975 bps was obtained and sequenced. Sequences were then submitted to the public database of Gene bank and NCBI under accession numbers, KY689139, KY689140, and KY689141. A Phylogenetic (Figure 3) tree of closely similar sequences of the *bsh* gene was constructed; sequences were obtained from BLAST conducted on NCBI.

#### 3.4.2 Cholesterol‐lowering assay

Cholesterol‐lowering activity or cholesterol reduction percentage of different strains of *L. acidophilus* has been presented in Table 5. All three strains possess the ability to reduce cholesterol from bile salts (0.2%) supplemented media with a maximum reduction of about 26% was observed after 24 hr of inoculation.

### 3.5 EPS extraction and quantification

Results of EPS production by strains as well as treatments (with time increase in EPS production) differed statistically as presented in Figure 3. Maximum EPS was produced after 24 hr of inoculation and incubation at 37℃ by *L. acidophilus* WFA1 (1.027 mg/ml) followed by WFA2 and WFA3 (0.83 and 0.685 mg/ml), respectively. Among all treatments (with time), WFA1 produced the highest amount of EPS at 6 hr (0.407 mg/ml), 12 hr (0.697 mg/ml), 18 hr (0.901 mg/ml), and 24 hr (1.027 mg/ml).

### 3.6 Comparison of antioxidant activity (DPPH, ABTS, and reducing power) of CFS of *L. acidophilus* strains with ascorbic acid

**FIGURE 4:**
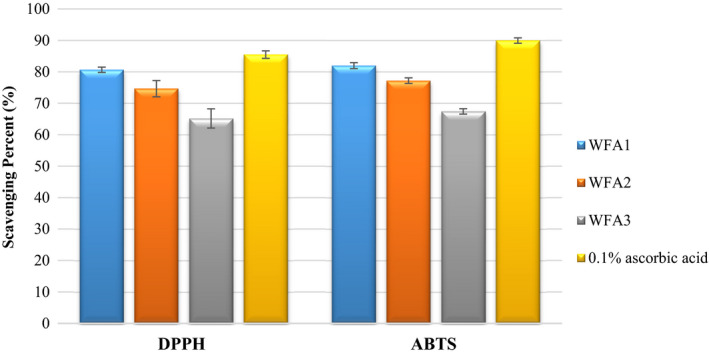
Comparison of DPPH and ABTS scavenging activity of *Lactobacillus acidophilus* strains after 24 hr of incubation with ascorbic acid

**FIGURE 5:**
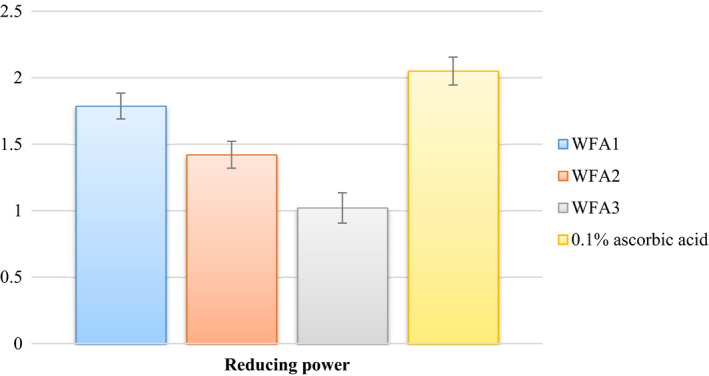
Comparison of reducing the power of *Lactobacillus acidophilus* strains after 24hrs of incubation with ascorbic acid

Antioxidant forms an irreversible or a stable complex by donating electrons or hydrogen atoms to the free radical (DPPH and ABTS accept electrons or hydrogen atoms from antioxidant substances and are converted into irreversible stable molecules). Comparison of DPPH, ABTS, and reducing power with ascorbic acid has been represented graphically in Figures 4 and 5, respectively. It was evident from data that DPPH scavenging activity of WFA1 (80.66%) has a nonsignificant difference from that of ascorbic acid (85.49%). ABTS scavenging activity (81.97%) and reducing power (1.78) of WFA1 have significantly differed from ascorbic acid (89.94%) and (2.05), respectively. Both of the other strains, that is, WFA2 and WFA3 have also high ABTS, DPPH, and reducing power but less when compared to the potential of WFA1.

## 4 DISCUSSION

To colonize in an intestinal portion of the alimentary tract, probiotic strain must tolerate and remain viable in harsh environmental conditions of the stomach (pH ranges from 1.5 to 3 or 4 and gastric enzymes) and intestine (bile and enzymes). It is obvious from earlier studies that this condition decreases bacterial viability by inhibiting their metabolism and growth (Liu et al., 2013). *Lactobacilli* have been reported previously for their resistance and survival under acidic (pH 2 and 3) (Sahadeva et al., 2011) and intestinal conditions (Pisano et al., 2014). *L. acidophilus* is also well documented for its resistance and survival in the stomach and intestine (Khaleghi et al., 2010).

In vitro cell surface hydrophobicity deals with the adhesion of probiotics to epithelial cell lining of the intestine, higher the hydrophobicity enhanced adhesion will be observed. On the basis of the degree of adhesion to hydrocarbons, Tyfa et al. (2015) categorized and divided bacterial strains into three categories including strongly hydrophobic (>50%), moderately hydrophobic (20%–50%), and hydrophilic (<20%). According to this criterion, all three strains of *L. acidophilus* falls in the category of strongly hydrophobic. In vitro bacterial adhesion to hydrocarbons was conducted against n‐hexadecane, xylene, and dichloromethane (Jose et al., 2015). Different studies reported different adherence percentages of *Lactobacilli*. Kaushik et al. (2009) studied the adherence percentage of probiotic *L*. *johnsonni* LA1 (47%), *L. acidophilus* LA7 (57%–58%), and *L*. *plantarum* (37.1%–37.7%). Similarly, Schillinger, Guigas, and Holzapfel, (2005) also reported bacterial cell surface hydrophobicity ranging from 74% to 95% in *L. acidophilus*. Among hydrocarbons used, xylene has shown a low mean value compared to dichloromethane and hexadecane that might be owing to its toxic or destructive behavior on microbial cells.

Bacterial auto‐aggregation capacity is considered as an aggregation among the cells of the same strains which further assessed their persistence in the intestine. Moreover, this aggregation eliminated or reduced the adhesion of pathogenic bacteria. Kaushik et al. (2009) reported cellular auto aggregation by *L. acidophilus* LA7 and *L*. *plantarum* (Lp9), which was about 46.5% and 31%, respectively. The results confirmed the self‐aggregating ability of *L. acidophilus*.

Cellular auto‐aggregation and SAT along with BATH collectively contributed to the cell surface hydrophobicity, which bacterial strains must possess to adhere to the intestinal mucosal or epithelial lining. Overall, adhesion of bacteria is mainly a strain‐specific property depending on its origin, variation in cell surface protein expression level along with the influence of environment on the expression of certain proteins which are responsible for cell surface hydrophobicity or cell adhesion (Chaffanel et al., 2018; De Vries et al., 2006). As bacterial adhesion to the human cell is a very complex process involving many components including charges on bacterial cell and human cell, hydrophobicity, the presence of extracellular polysaccharide and cell surface proteins (Pan et al., 2017), therefore for proper, strong or irreversible adhesion, the bacterial cells have to overcome all of these hurdles. Adhesion property increases as the nonpolarity of both or one of the surfaces (microbial and host cell) increases.

Strains of LAB have been widely studied previously for their hypocholesterolemic activity both in vivo and in vitro. One of the probable mechanisms for this was the deconjugation of bile by Bsh. Bsh activity involves enzymatic breakdown or deconjugation of bile salts or bile acids by probiotic *Lactobacilli* and *Bifidobacterium*, increasing its excretion or a decrease in its reabsorption. Cholesterol, being a precursor of bile acids, converts its molecules to bile acids as compensation or to replace lost ones, thus decreasing the cholesterol level. In the control of serum cholesterol levels, this mechanism could be operated by conversion of deconjugated bile acids by colonic microbes (Patel et al., 2010). Bsh activity was supposed to be the important criteria as nondeconjugating organisms do not appear to be able to remove cholesterol from the culture medium to any significant extent (Kumar et al., 2012; Tahri et al., 1997).

Moreover, Gilliland et al. (1985) reported cholesterol removal by *L. acidophilus* strains from culture medium when grown under simulated intestinal conditions. As this is a strain‐dependent property, therefore in different studies the cholesterol‐lowering percentage differs significantly (Lye et al., 2010). Lim et al. (2004) reported about 31.5%–58.5% reduction, and Sirilun et al. (2010) observed 25.41 to 81.46% reduction in cholesterol. Similarly, about 26.74%–85.41% reduction of cholesterol from the growth medium after 20 hr of inoculation of different *Lactobacilli* strains was reported by Ramasamy et al. (2010).

Our results also confirm that there is a relation between bile salt hydrolase activity and the cholesterol‐lowering effect. Strains having Bsh activity also can reduce cholesterol levels from serum (Öner et al., 2014). *Lactobacilli* have also been reported previously for their cholesterol‐lowering attributes. Similar to our study, Jones et al. (2004) evaluated the cholesterol‐lowering potential of LAB and attributed this to bsh activity, due to deconjugation of conjugated glycodeoxycholic acid and taurodeoxycholic acid. Reduction of cholesterol by LAB and *Bifidobacteria* in the presence of bile salts were also been reported in previous researches (Huang et al., 2019; Tsai et al., 2014).

Quantification of EPS was conducted in many studies where Deepak et al. (2016a) reported about 400–597 mg/L of EPS produced by *L. acidophilus* after 24 hr of incubation under optimized conditions. Similarly, Polak‐Berecka et al. (2014) reported about 210.28 mg/L of EPS by *L*. *rhamnosus* E/N strain. EPS are polymers of sugars, arranged as long‐chained polysaccharides, while their functional properties may be due to the arrangement of monosaccharides, as well as the type and conformation of glycosidic linkages of the polysaccharide (Suresh Kumar et al., 2007). EPS of LAB are used in food to enhance its taste and texture, ability to operate as an emulsifier, and maintaining viscosity (bio stabilizer) of fermented foods. Apart from this, EPS also have strong antioxidant, anticancer, and anti‐inflammatory activities (Deepak, Ramachandran, et al., 2016; Liu et al., 2011).

Similarly, numerous researches were conducted on DPPH scavenging ability, ABTS cation radical scavenging potential, and reducing the power of CFS of LAB, respectively, and they also reported its high antioxidant potential (Afify et al., 2012; Gao, 2012; Xing et al., 2015). Mostly 0.05% of the ascorbic acid concentration was used previously in different studies. In the present study, we used an almost double percentage of ascorbic acid, that is, 0.1%. Ji et al. (2015) reported high DPPH and ABTS scavenging activity of CFS of *Lactobacilli* when compared to 0.5% ascorbic acid (that was considered as a high concentration of ascorbic acid). Furthermore, (Liang et al., 2016) also used ascorbic acid as a positive control or standard for comparison and reported high antioxidant potential. The antioxidant potential of different microbial or bacterial strains can be attributed to the production of their cell surface compounds, for example, the production of exo‐polysaccharide (Oh & Jung, 2015) and lipoteichoic acid (Yi et al., 2009).

## 5 CONCLUSION

Strains of *L. acidophilus* (WFA1, WFA2, and WFA3) have resisted and survived under GI tract conditions, furthermore; they can adhere and colonize in the intestinal cavity, thereby confirming their probiotic potential. These strains also have cholesterol‐lowering properties along with high EPS production and antioxidant activity. Among the three strains, WFA1 was selected further due to its potential functional properties and was supplemented to produce probiotic fermented milk.

## CONFLICT OF INTEREST

The authors declared that there is no conflict of interest.

## AUTHOR CONTRIBUTIONS

**Wajiha Farid :** Conceptualization (equal); Data curation (equal). **Tariq Masud:** Supervision (equal). **Asma Sohail:** Methodology (equal). **Nazir Ahmad:** Data curation (equal). **S.M. Saqlan Naqvi:** Visualization (equal). **Sipper Khan :** Software (equal). **Amjad Ali:** Supervision (equal). **Salah Khalifa:** Writing‐original draft (equal). **ABID HUSSAIN:** Resources (equal). **Sartaj Ali:** Validation (equal). **Maryum Saghir:** Formal analysis (equal). **Azhari Siddeeg:** Resources (equal). **Muhammad Faisal Manzoor:** Formal analysis (equal); Writing‐review & editing (equal).

## Data Availability

The dataset supporting the conclusions of this article is included within the article.
